# Efficacy and Safety Profile of Montgomery T-Tube Implantation in Patients with Tracheal Stenosis

**DOI:** 10.1155/2020/2379814

**Published:** 2020-10-07

**Authors:** Feng-Jie Wu, Yang-Wei Yao, En-Guo Chen, Hui-Hui Hu, Jian-Ping Jiang, Meng Yang, Yang-Yang Gu, Da-Kui Cao, Ye-Li Zhu

**Affiliations:** ^1^Department of Respiratory Medicine, The Second Affiliated Hospital of Jiaxing University, Jiaxing, Zhejiang, China; ^2^Department of Respiratory Medicine, Sir Run Run Shaw Hospital, College of Medicine, Zhejiang University, Hangzhou, Zhejiang, China

## Abstract

**Background:**

Tracheal stenosis is able to lead to airway obstruction.

**Objective:**

To evaluate the efficacy and safety profile of Montgomery T-tube implantation in patients with tracheal stenosis.

**Methods:**

Fifty-two patients with tracheal stenosis diagnosed between 2016 and 2019 were included in this retrospective cohort study. The patients were divided into observation group (*n* = 25 cases) and control group (*n* = 27). The therapeutic effect, arterial blood gas analysis, arterial oxygen partial pressure (PaO_2_), arterial carbon dioxide partial pressure (PaCO_2_), shortness of breath score, airway diameter change, dyspnea score, quality of life, and safety were compared between the two groups before and after treatment.

**Results:**

The therapeutic effect of the observation group gained better results than that of the control group (84.00% vs. 62.96%). One week after operation, the pH value, SaO_2_, PaCO_2_, shortness of breath score, airway diameter change, dyspnea score, life quality, and incidence of postoperative complications in the observation group exerted better results as compared to the control group.

**Conclusion:**

The implantation of Montgomery T-tube has effective function in terms of improving the symptoms of dyspnea and the life quality of patients with safety profile in patients harboring tracheal stenosis.

## 1. Introduction

Tracheal stenosis is able to lead to airway obstruction, causing shortness of breath and dyspnea, which is aggravated by increased respiratory secretions, physical activity, or aerobic exercise, accompanied by wheezing and can result in asphyxia in serious cases [1, 2]. Tracheal stenosis poses a serious threat towards the life and safety of patients with high disability rate and mortality. It is currently difficult to treat, and finding the appropriate early intervention is highly recommended. In clinical practice, the disease can be divided into benign and malignant, in which the main causes of benign stenosis include congenital malformation, infection, and traumatic injury. And the main causes of malignant stenosis are the compression of malignant lesions in the surrounding tissue and primary malignant lesions of the lung and trachea [3]. Nowadays, the main approaches in terms of treating tracheal stenosis include tracheal reconstruction and surgery. However, considering the systemic complications, the existence of serious organ stenosis, and severe tracheal injury, several patients are unable to have surgical interventions. There are also patients suffering from central nervous system injury and other conditions who need to recover before surgical treatment. Hence, it is necessary to maintain the patency of the abovementioned patients' airway [4]. In recent years, with the advanced progress of medical technology, respiratory interventional therapy and interventional technology under bronchoscope also experience remarkable development. Surgical approaches are widely used with good outcomes, such as balloon argon knife under bronchoscope, freezing, dilatation, etc. Nevertheless, there are some patients who do not achieve the beneficial outcomes. Based on the study by Prasanna Kumar et al. [5], Montgomery T-type silicone tube is effective in the treatment of tracheal stenosis. Therefore, in an attempt to investigate the clinical effect of Montgomery T-tube implantation in patients with tracheal stenosis, 52 patients harboring tracheal stenosis were studied in our hospital from January 2016 to January 2019.

## 2. Methods

### 2.1. Participants

A total of 52 patients with tracheal stenosis, admitted to our hospital from January 2016 to January 2019, were divided into observation group and control group. In the observation group (*n* = 25), there were 18 males and 7 females, aged 47–75 years, with an average age of 63.01 ± 6.87 years, and 4 patients who have undergone bronchotomy, 9 patients with traumatic scar after endotracheal intubation or tracheotomy and 12 patients with tuberculous bronchial scar were included. In the control group (*n* = 27), there were 19 males and 8 females, aged 46–74 years, with an average age of (62.76 ± 7.01) years, including 5 patients after sleeve bronchotomy, 10 patients with traumatic scars after tracheal intubation or tracheotomy, and 12 patients with tuberculous bronchial scars. The main clinical manifestations of all the above patients were dyspnea, cough, expectoration, and with a history of tracheotomy. The general data of the two groups were comparable with no significant difference (*P* > 0.05).

### 2.2. Treatment Approach

Before operation, all patients stopped taking anticoagulants and antiplatelet drugs, fasted at the same time, and were required to complete treatment-related examinations. Focus had been laid on clotting time, platelets, and so on. The location, scope, and degree of stenosis were evaluated according to the results of chest CT examination, and general anesthesia was used. Patients in the control group were given tracheal dilatation via fiberoptic bronchoscope. Supine position was taken and pillow was removed, and tracheal balloon entered the narrow segment of trachea and bronchus through bronchoscope operation hole. After the balloon in tracheal balloon was placed at both ends of the narrow segment under fiberoptic bronchoscope, the balloon was injected with sterilization water from low to high using gun pump with 100–400 kPa pressure. The balloon should be kept expanding for no more than 1 min when inflated for the first time. Bleeding should be observed, and if there was no bleeding, the abovementioned process should be performed for 3 times to keep the balloon expansion time of 1–3 min. If the diameter of the trachea increased with balloon dilatation, the operation should be considered successful; if the diameter of the trachea did not dilate with the balloon, the balloon dilatation should be performed again 7 days later.

**Figure 1:**
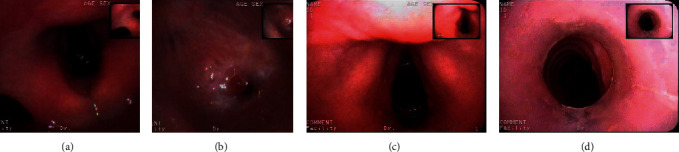
Airway and glottic status before and after surgery. (a) The preoperative hilum; (b) the preoperative airway; (c) the postoperative glottis; and (d) the postoperative airway.

The patients in observation group were treated with Montgomery T-tube implantation. Firstly, the airway was established according to the results of chest CT, and the length and diameter of Montgomery T-tube were calculated based on the diameter of the normal part of the airway (measured by tracheoscope) and the results of airway reconstruction. In clinical practice, the diameter of Montgomery T-tube is usually 11 mm to 14 mm, and its length should be at least about 3 mm longer than that of both ends of tracheal stenosis, and its proximal end should be 0.4 cm under subglottic area. With the laryngeal mask ventilation for general anesthesia, the fiberoptic bronchoscope entered the trachea to observe the specific conditions in the lower tracheal lumen. After the narrow segment of the trachea was treated with high-frequency electric knife and balloon dilatation, the air-cut cannula was removed. After curling the end of the Montgomery T-tube, the curled end of the Montgomery T-tube was clamped with a curved vascular clamp and placed in the trachea through the tracheal incision, and continued to push forward until it was completely into the trachea. In order to make its end into the subglottic trachea above the air incision, the side limbs of the Montgomery T-tube should be pulled to complete the placement of the Montgomery T-tube, and the placement of the Montgomery T-tube should be observed by fiberoptic bronchoscope (Figure 1).

During the above operation, if the patient was complicated with local granulation tissue hyperplasia, high-frequency electric knife cauterization should be performed during the operation; if the tracheal wall was softened, a nitinol stent may be inserted intraoperatively. After the operation, all the patients received intraluminal infusion of antibiotics to prevent infection and inhaled budesonide (trade name: inhalation budesonide suspension; specification: 60 inhalations (160 ug: 4.5 ug); manufacturer: AstraZeneca AB; approval no. H20140475) for 3 to 6 consecutive months.

### 2.3. Observation Indicators

The therapeutic effects and intraoperative complications of the two groups were observed, and the indexes of blood gas analysis, shortness of breath score, changes of airway diameter, dyspnea score, and quality of life were compared between the two groups before treatment and one week after operation.

### 2.4. Therapeutic Effect

Therapeutic effect [6] included the following aspects: (1) failed: the initial reexpansion of the airway was not successful; (2) ineffective: the initial reexpansion of the airway was successful, but the caliber was unstable, and the treatment interval was no more than 3 months or there was occlusion in final airway; (3) effective: the initial reexpansion of the airway was successful, but the caliber of reexpansion was unstable, and the treatment interval was more than 6 months; and (4) cured: the initial airway reexpansion was successful, the reexpansion caliber was stable, and the treatment interval was more than 1 year. Total effective rate = cure rate + effective rate.

Blood gas analysis index included pH value, blood oxygen saturation (SaO_2_), arterial carbon dioxide partial pressure (PaCO_2_).

### 2.5. Shortness of Breath Score

According to the shortness of breath rating standard of the American Thoracic Association [7], it was divided into 4 grades: level 0: patients with shortness of breath during slight activity; level 1: patients stop walking due to shortness of breath when walking at normal speed; level 2: patients with shortness of breath when walking at normal speed; level 3: patients with shortness of breath during brisk walking; and level 4: normal.

### 2.6. Dyspnea Score

Based on the mMRC grading method [8], the score was divided into 4 levels: level 0: patients having obvious symptoms of dyspnea, unable to leave the room, or with shortness of breath in the process of wearing and taking off clothes; level 1: patients have to rest after walking for a few minutes or 100 m on flat ground; level 2: compared with people of the same age, patients walk slower due to dyspnea, or need to rest in the walking process on flat ground; level 3: patients with shortness of breath in the process of fast walking and going up a gentle slope; and level 4: there are no obvious symptoms of dyspnea unless strenuous exercise.

### 2.7. Quality of Life

WHOQOL-BREF [9] was used to evaluate the patients' quality of life, with a full score of 100. Higher score represented the better life quality of patient.

### 2.8. Safety Profile

The occurrence of intraoperative complications was recorded.

### 2.9. Statistical Analysis

All data in this study were analyzed by SPSS18.0 software, the measurement data were expressed by $\left(\bar{x}\pm s\right)$ and *t*-test, and the counting data were expressed by the number of cases and percentage and accurately tested by *x*^2^-test or Fisher. A *P* < 0.05 represented significant difference.

## 3. Results

### 3.1. Comparison of Therapeutic Effects between the Two Groups

The therapeutic effect of the observation group was better than that of the control group (84.00% vs. 62.96%) (*P* < 0.05), as laid out in Table 1.

### 3.2. Comparison of Blood Gas Analysis Indexes between the Two Groups

Before treatment, there was no significant difference in pH, SaO_2_, and PaCO_2_ between the two groups (*P* > 0.05). One week after operation, the pH and levels of SaO_2_ and PaCO_2_ were improved in both groups, and the pH and levels of SaO_2_ and PaCO_2_ in the observation group were better than those in the control group (*P* < 0.05), as shown in Table 2.

### 3.3. Comparison of Shortness of Breath Score between the Two Groups

**Figure 2:**
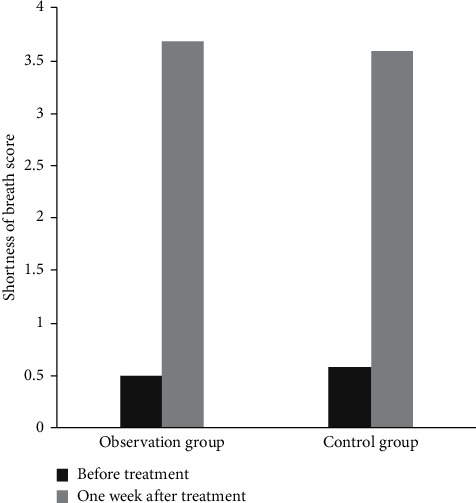
Comparison of shortness of breath score between the two groups. Note: compared with the same group before treatment (^a^*P* < 0.05) and the control group after treatment (^b^*P* < 0.05).

Before treatment, there was no significant difference in the score of shortness of breath between the two groups (*P* > 0.05). One week after operation, the score of shortness of breath was improved in both groups, and the score of shortness of breath in the observation group was significantly higher than that in the control group (*P* < 0.05), as shown in Figure 2.

### 3.4. Comparison of the Changes of Airway Diameter between the Two Groups

**Figure 3:**
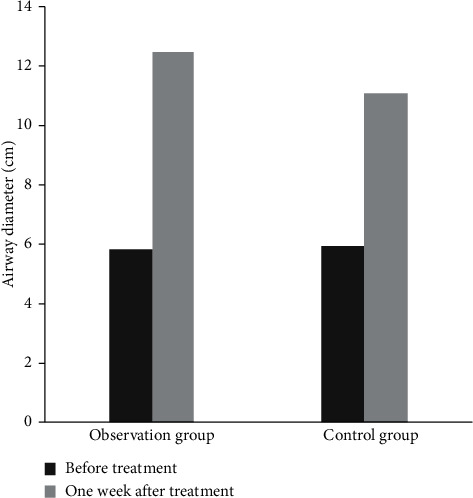
Comparison of the changes of airway diameter between the two groups. Note: compared with the same group before treatment (^a^*P* < 0.05) and the control group after treatment (^b^*P* < 0.05).

Before treatment, there was no significant difference in the change of airway diameter between the two groups (*P* > 0.05). One week after operation, the airway diameter was increased in both groups, of which the change of airway diameter in the observation group was more obvious than that in the control group (*P* < 0.05), as shown in Figure 3.

### 3.5. Comparison of Dyspnea Scores between the Two Groups

**Figure 4:**
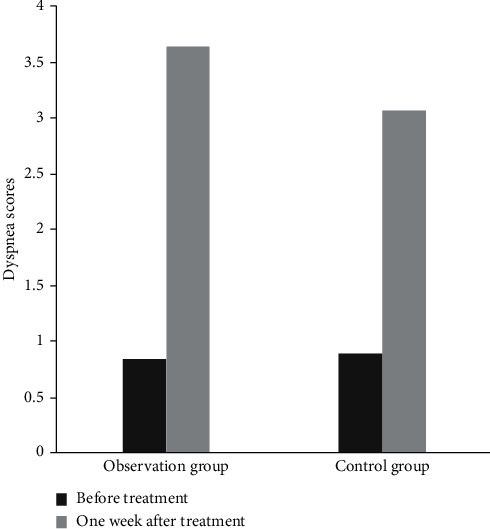
Comparison of dyspnea scores between the two groups. Note: compared with the same group before treatment (^a^*P* < 0.05) and the control group after treatment (^b^*P* < 0.05).

Before treatment, there was no significant difference in dyspnea score between the two groups (*P* > 0.05). One week after operation, the dyspnea score was improved in both groups, of which the dyspnea score in the observation group was significantly higher than that in the control group (*P* < 0.05), as shown in Figure 4.

### 3.6. Comparison of Quality of Life between the Two Groups

**Figure 5:**
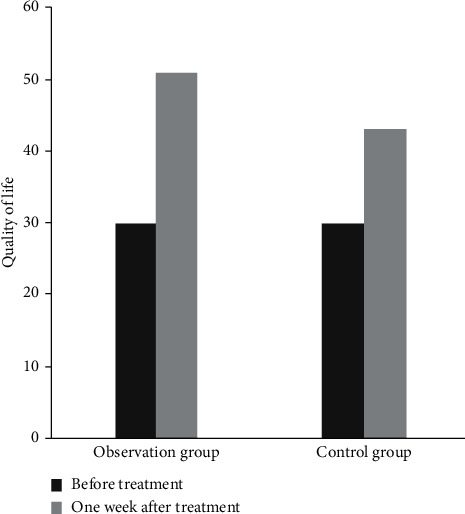
Comparison of quality of life between the two groups. Note: compared with the same group before treatment (^a^*P* < 0.05) and the control group after treatment (^b^*P* < 0.05).

Before treatment, there was no significant difference in the quality of life between the two groups (*P* > 0.05). One week after operation, however, the quality of life was improved in both groups, and the life quality in the observation group was significantly higher than that in the control group (*P* < 0.05), as shown in Figure 5.

### 3.7. Comparison of Intraoperative Complications between the Two Groups

In the current study, 4 patients in the control group received balloon treatment for the first time, and the operation failed. One week later, 2 patients were operated on successfully, and 2 failed in the second operation. Montgomery T-tube implantation was used. In the other 6 patients, there were tearing airways. And 2 cases had massive hemorrhage (the amount of bleeding was more than 150 ml). In the observation group, there were 2 cases of displacement of Montgomery T-tube and 2 cases of subcutaneous emphysema after replacement. The incidence of complications in the observation group was significantly lower than that in the control group (8.00% vs. 22.22%) (*P* < 0.05).

## 4. Discussion

Airway stenosis is one of the most common diseases in pneumonology, caused by pathological changes of the airway wall following various etiological factors. Its main clinical symptoms include dyspnea, decreased activity endurance, accompanied with dry inspiratory rales, which can lead to suffocation and death in severe cases, seriously affecting the quality of life of patients [10, 11]. In clinical practice, the diagnosis is determined by chest CT or tracheoscopy combined with medical history and symptoms. For some patients with tracheal stenosis, lesions may not be completely removed, and improper suture at the end of the operation will lead to restenosis of the tracheal scar. The degree and length of stenosis affect the choice of operation. Currently, with the continuous progress of the treatment technology under the tracheoscope, the technology under the fiberoptic bronchoscope experiences great development. The popularization and application of balloon dilatation, high-frequency electric knife, laser, and other treatment approaches remarkably improve the clinical symptoms of most patients. But there are some other patients who suffer from poor outcomes. The implantation of metal stent can alleviate the clinical symptoms of patients, but there will be higher incidence of complications, such as stent fracture, stent displacement, and granulation tissue proliferation. Obviously, metal stent has several limitations [12]. In 1968, the Montgomery T-tube invented by Montgomery was primarily invented to maintain normal ventilation after surgical airway reconstruction or during tracheal stenosis surgery [13]. Several studies [5, 14] have confirmed that with the continuous improvement of Montgomery T-tube, it has good histocompatibility with patients. Now, it has become an important tool for the treatment of tracheal diseases and can be used as an independent treatment. The Montgomery T-type stent has the following advantages: (1) it is made of medical silicone, the texture is soft, the stimulation to the airway is negligible, it is unlikely for granulation to grow at both ends of the stent, and it is prominently used in the subglottic part; (2) Montgomery T-type stent has good support, and the airway recovery and shaping effect are satisfactory after selecting a suitable model; (3) it has good stability and can avoid moving in the airway; and (4) it is beneficial for patients to resume oral ventilation, speak, and have better comfort compared with tracheotomy.

According to the results of the current study, compared with the control group, the observation group elicited greater beneficial outcomes in terms of therapeutic effect and blood gas analysis index (*P* < 0.05). It was suggested that Montgomery T-tube implantation can effectively improve the therapeutic effect and improve the blood gas analysis index of patients with tracheal stenosis. In our study, Montgomery T-tubes were implanted through fiberoptic bronchoscope under general anesthesia, and all patients were successfully performed. Most patients were with grade II Cotton–Myer or below, only 3 patients were with grade III Cotton–Myer, but all of them were placed successfully. Based on the author's clinical experience of more than ten years, patients with grade III Cotton–Myer tracheal stenosis should be treated with comprehensive interventional therapy under general anesthesia after the Montgomery T-tube was placed in. If the inner segment of the Montgomery T-tube cannot be completely dilated, the balloon-assisted Montgomery T-tube should be used to open the inner segment of the Montgomery T-type stent. The results of this study showed that one week after operation, the score of shortness of breath, the change of airway diameter, the score of dyspnea, and the quality of life in the observation group were better than those in the control group (*P* < 0.05), indicating that the implantation of Montgomery T-tube in patients with tracheal stenosis can effectively improve the outcomes of patients with shortness of breath and dilate the airway diameter, which is beneficial to breathing and improves the quality of life of patients. Subcutaneous emphysema, no secretion retention, granuloma, and local skin infection after Montgomery T-tube implantation are easy to appear. Based on the results of complications in this study, only 2 cases of subcutaneous emphysema were found in the observation group, which was closely related to the shallow position of Montgomery T-tube implantation. After deep placement, the inner segment of the tube was not long enough, it was reimplanted after recalculation, and the subcutaneous emphysema disappeared. Therefore, the accurate length and diameter of Montgomery T-tube must be calculated before placing the tube. In the treatment of balloon dilatation, due to the large balloon dilatation pressure needed during the operation for a certain period of time, with mechanical ventilation being unable to be carried out at the same time as high-pressure tracheal balloon dilatation, so patients need to hold their breath for a long time and affect the tolerance and expansion time. High pressure will easily result in tearing airway and cause massive bleeding; hence, attention should be paid to the safety of the patients. In the present study, there were 6 cases of airway tear in the control group, of which 2 cases had massive hemorrhage. The patient's airway tear may be related to high intraoperative pressure. When the patient's airway is severely torn, massive hemorrhage may occur, which does not require special treatment. The patient's clinical manifestation can be continuously observed, and local adrenaline treatment can be performed if necessary. The results of this study showed that the incidence of complications in the observation group was significantly lower than that in the control group (*P* < 0.05), indicating that it is safe to use Montgomery T-tube implantation in patients with tracheal stenosis.

At present, there is no final conclusion on the time of Montgomery T-tube implantation. According to several earlier clinical studies, the period for patients with the stent should be controlled at 6 to 24 months [15]. However, after extubation, the same type of metal casing should be used, and the replaced metal casing should be blocked for 24 hours. If the patient is able to normally breathe within 2 weeks, the metal casing can be removed. However, the author of this study believes that focus should be laid on the specific conditions of the patients. In the observation group, 3 patients gained improved clinical symptoms 6 months after operation, and the trachea returned to normal based on airway endoscopy. The Montgomery T-tube was removed successfully. Considering the limited sample size of this study, more studies with larger sample size are required in order to further explore the postoperative voice quality and complications of patients after operation.

## 5. Conclusion

Montgomery T-tube implantation is an effective approach in terms of improving the clinical symptoms and the life quality in patients harboring tracheal stenosis with safety profile, which is considered available for wide clinical use.

## Figures and Tables

**Table 1:** Comparison of therapeutic effects between the two groups (%).

Group	Failed	Ineffective	Significantly effective	Cured	Efficiency
Observation group (*n* = 25)	0	4 (16)	7 (28.00)	14 (56.00)	21 (84.00)^b^
Control group (*n* = 27)	2 (7.41)	8 (29.63)	9 (33.33)	8 (29.63)	17 (62.96)

Note: compared with the control group (^b^*P* < 0.05).

**Table 2:** Comparison of blood gas analysis indexes between the two groups ($\bar{x}\pm s$, %).

Group	Time	pH value (mmHg)	SaO_2_ (mmHg)	PaCO_2_ (mmHg)
Observation group (*n* = 69)	Before treatment	7.27 ± 0.12	83.09 ± 7.81	57.91 ± 5.41
	One week after operation	7.39 ± 0.18^a, b^	98.03 ± 8.91^a, b^	40.71 ± 8.62^a, b^
	Difference before and after treatment	0.12 ± 0.06	14.94 ± 1.10	−17.20 ± 3.21

Control group (*n* = 69)	Before treatment	7.26 ± 0.62	83.87 ± 8.73	57.03 ± 5.19
	One week after operation	7.35 ± 0.92^a^	95.42 ± 9.01^a^	43.82 ± 8.38^a^
	Difference before and after treatment	0.09 ± 0.30	11.550.28±	14.21 ± 3.14

Note: compared with the same group before treatment (^a^*P* < 0.05) and the control group after treatment (^b^*P* < 0.05).

## Data Availability

The data used to support the findings of this study are available from the corresponding author upon reasonable request.

## Abbreviations

PaCO_2_: Arterial carbon dioxide partial pressure
PaO_2_: Arterial oxygen partial pressure.
